# Supplementary material for: Ultrafast-Laser Micro-Structuring of LiNi0.8Mn0.1Co0.1O2 Cathode for High-Rate Capability of Three-Dimensional Li-ion Batteries
Source: Nanomaterials (Basel). 2022 Nov 4;12(21):3897. doi: 10.3390/nano12213897 (PMC9654857; doi:10.3390/nano12213897)
Supplement: Supplementary file 1 [file nanomaterials-12-03897-s001.zip › nanomaterials-1990605-supplementary.pdf]

nanomaterials

MDPI

Supplementary Material

Ultrafast-Laser Micro-Structuring of $LiNi_{0.8}Mn_{0.1}Co_{0.1}O_2$ Cathode for High-Rate Capability of Three-Dimensional Li-ion Batteries

Minh Xuan Tran [1,2], Peter Smyrek [3,4], Jihun Park [5], Wilhelm Pfleging [3,4,]* and Joong Kee Lee [1,2,]*

1 Division of Energy and Environmental Technology, KIST School, Korea University of Science and Technology (UST), Deajeon 34113, Korea
2 Center for Energy Storage Research, Green City Research Institute, Korea Institute of Science and Technology (KIST), Seoul 02792, Korea
3 Karlsruhe Institute of Technology, Institute for Applied Materials, P.O. Box 3640, 76021 Karlsruhe, Germany
4 Karlsruhe Nano Micro Facility, Hermann-von-Helmholtz-Platz 1, 76344 Eggenstein-Leopoldshafen, Germany
5 APC Technology, 108 68 Gangbyeonyeok-ro-4-gil, Gwangjin-gu, Seoul 05116, Korea
* Correspondence: wilhelm.pfleging@kit.edu (W.P.); leejk@kist.re.kr (J.K.L.)

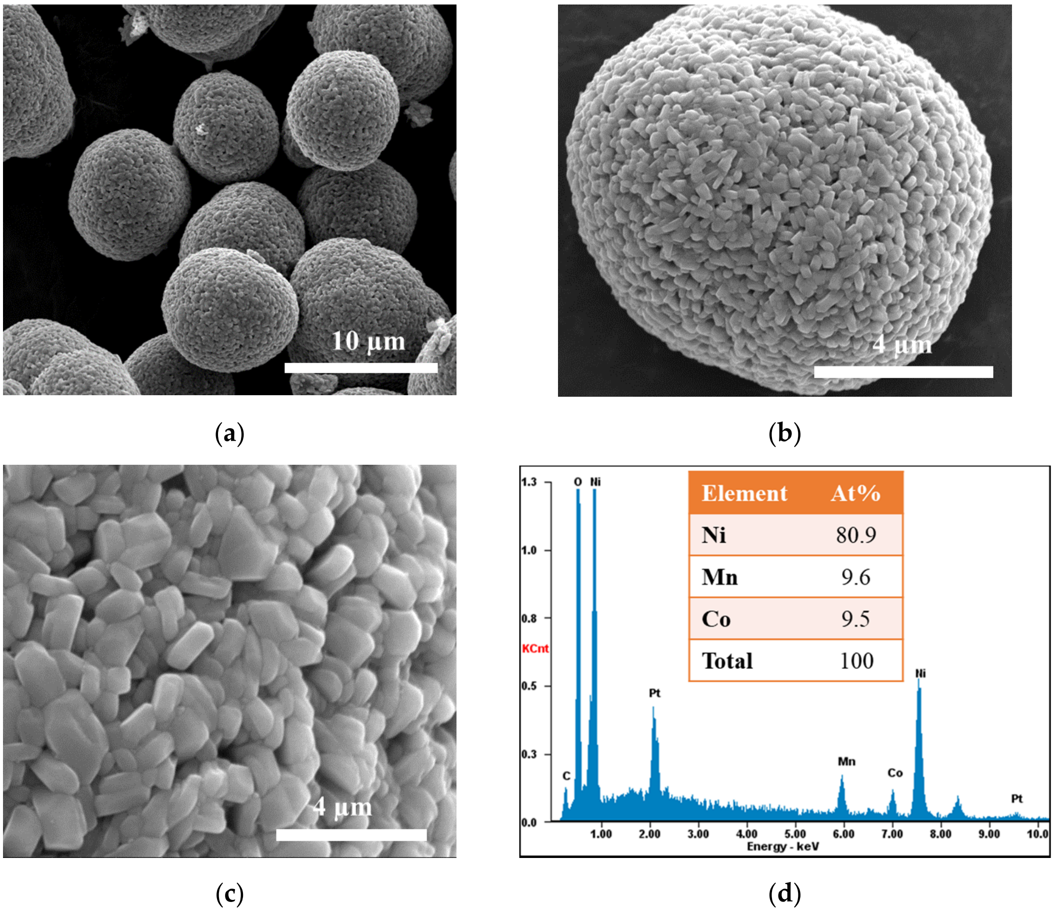

Figure S1. Morphology of NMC811 particles. ((a, b, c) SEM images of NMC811 particles and (d) EDX spectrum and element composition of selected area from SEM image (Figure c)**.**

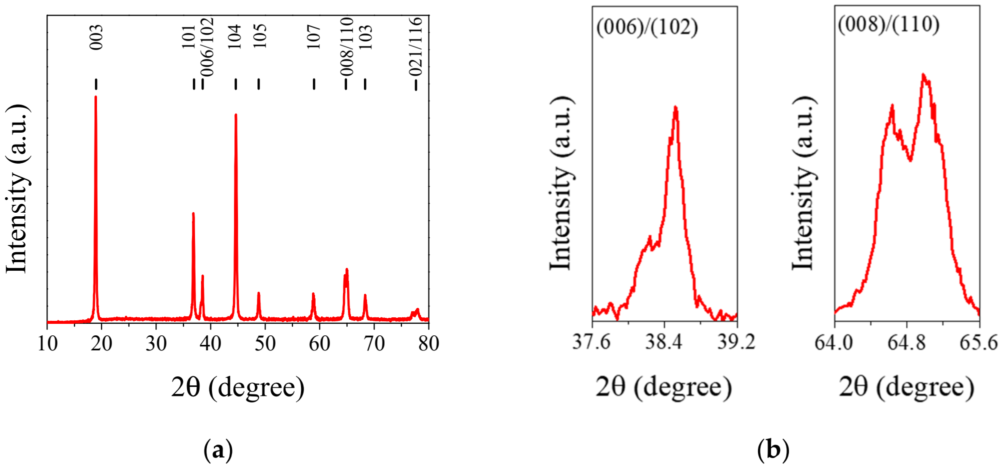

Figure S2. (a) XRD patterns of NMC811 and (b) the enlarged diffraction of (006)/(102) and (008)/(110) XRD peaks.

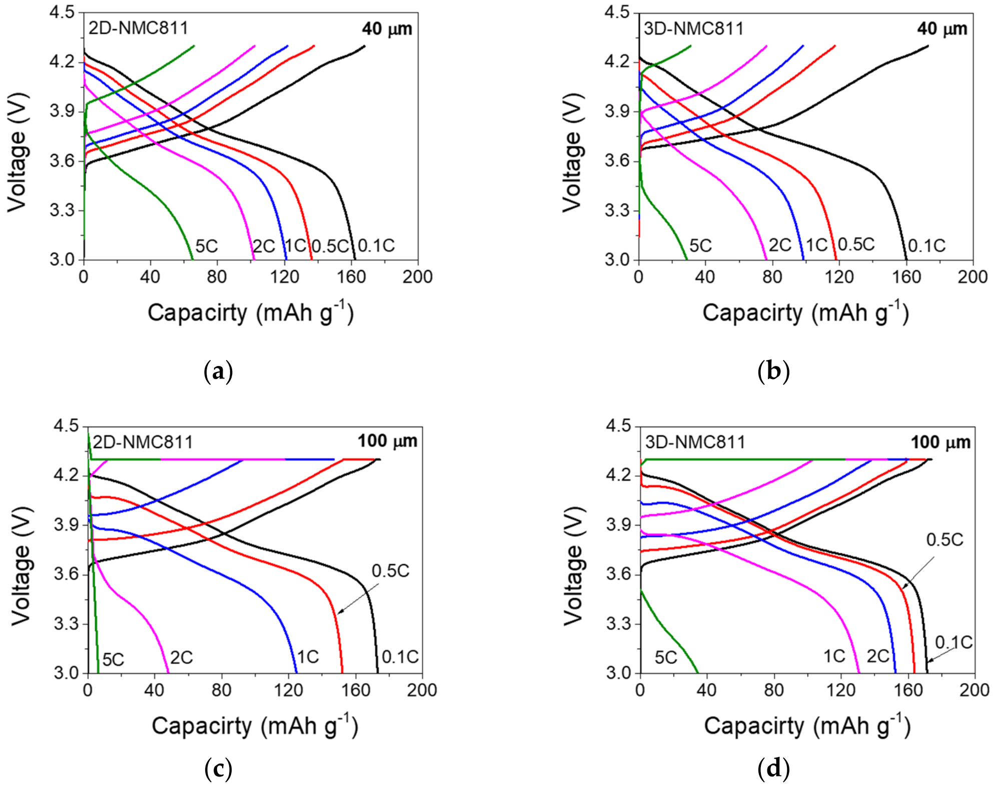

Figure S3. Galvanostatic charge/ discharge profiles of (a) 40-μm-2D-NMC811, (b) 40-μm-3D-NMC811, (c) 100-μm-2D-NMC811, and (d) 100-μm-3D-NMC811.

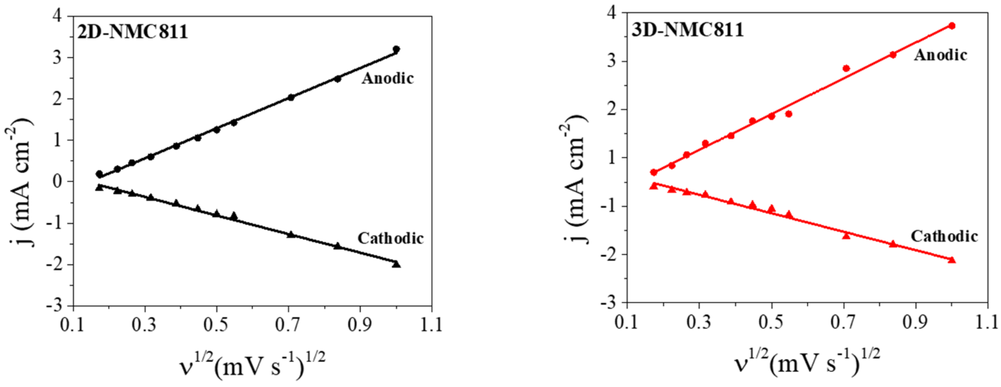

(**a**) (**b**)

Figure S4. Diffusivity coefficient calculation. Normalized peak current obtained from CV scans plotted as a function of the square root of the scan rate for (a) 2D-NMC811 and (b) 3D-NMC811.

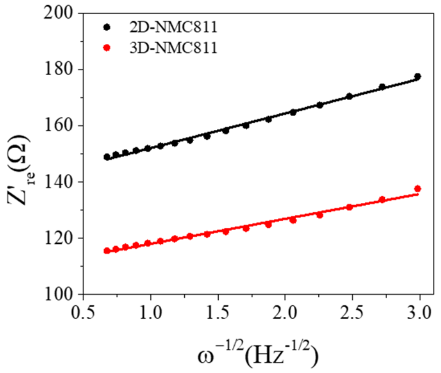

Figure S5. Linear fit lines demonstrating correlation between Z_{re} and $\omega^{-1/2}$ extracted from EIS data.

Table S1. Summary of effect of laser structuring on electrodes at various thickness. .

**Characteristics**	**Electrode thickness (μm)**	
	**40**	**100**
Increase of interfacial area (%)	20	78
Aspect ratio (*AR*)	0.96	3.72
Loss of material (%)	10	6.4
Effective area (cm^2)	1.13	2.01

Table S2. Fitting results for impedance spectra and calculated D_{Li+} from CVs and electrochemical impedance spectroscopy.

**Electrodes**	**D_{Li+} (cm s^{-2}) by CVs**		**EIS**		
	**Anodic**	**Cathodic**	**R_s (Ω)**	**R_{CT} (Ω)**	**D_{Li+} (cm s^{-2})**
2D-NMC811	16.6 × 10^{-11}	2.0 × 10^{-11}	3.2	136.6	1.6 × 10^{-11}
3D-NMC811	18.9 × 10^{-11}	1.9 × 10^{-11}	1.4	107.7	3.5 × 10^{-11}
